# *Catharanthus roseus *flower extract has wound-healing activity in Sprague Dawley rats

**DOI:** 10.1186/1472-6882-6-41

**Published:** 2006-12-21

**Authors:** BS Nayak, Lexley M Pinto Pereira

**Affiliations:** 1 Department of Pre Clinical Sciences, Biochemistry Unit, Faculty of Medical Sciences, The University of the West Indies, St. Augustine, Trinidad; 2 Department of Para Clinical Sciences, Pharmacology Unit, Faculty of Medical Sciences, The University of the West Indies, St. Augustine, Trinidad

## Abstract

**Background:**

*Catharanthus roseus *L (*C. roseus*) has been used to treat a wide assortment of diseases including diabetes. The objective of our study was to evaluate the antimicrobial and wound healing activity of the flower extract of *Catharanthus *in rats.

**Methods:**

Wound healing activity was determined in rats, after administration (100 mg kg^-1 ^day^-1^) of the ethanol extract of *C. roseus *flower, using excision, incision and dead space wounds models. The animals were divided into two groups of 6 each in all the models. In the excision model, group 1 animals were topically treated with carboxymethyl cellulose as placebo control and group 2 received topical application of the ethanol extract of *C. roseus *at a dose of 100 mg/kg body weight/day. In an incision and dead space model group 1 animals were given normal saline and group 2 received the extract orally at a dose of 100 mg kg^-1 ^day^-1^. Healing was assessed by the rate of wound contraction, period of epithelization, tensile strength (skin breaking strength), granulation tissue weight, and hydoxyproline content. Antimicrobial activity of the flower extract against four microorganisms was also assessed

**Results:**

The extract of *C. roseus *significantly increased the wound breaking strength in the incision wound model compared with controls (P < 0.001). The extract-treated wounds were found to epithelialize faster, and the rate of wound contraction was significantly increased in comparison to control wounds (P < 0.001), Wet and dry granulation tissue weights, and hydroxyproline content in a dead space wound model increased significantly (p < 0.05). *Pseudomonas aeruginosa *and *Staphylococcus aureus *demonstrated sensitivity to *C. roseus*

**Conclusion:**

Increased wound contraction and tensile strength, augmented hydroxyproline content along with antimicrobial activity support the use of *C. roseus *in the topical management of wound healing.

## Background

The therapeutic efficacies of many indigenous plants, for various diseases have been described by traditional herbal medicine practitioners [1]. Natural products are a source of synthetic and traditional herbal medicine. They are still the primary health care system in some parts of the world [2]. The past decade has seen considerable change in opinion regarding ethnopharmacological therapeutic applications. The presence of various life sustaining constituents in plants has urged scientists to examine these plants with a view to determine potential wound healing properties.

Wound healing is the process of repair that follows injury to the skin and other soft tissues. Following injury, an inflammatory response occurs and the cells below the dermis (the deepest skin layer) begin to increase collagen (connective tissue) production. Later, the epithelial tissue (the outer skin layer) is regenerated. There are three stages to the process of wound healing: inflammation, proliferation, and remodeling. The proliferative phase is characterized by angiogenesis, collagen deposition,, epithelialisation and wound contraction. Angiogenesis involves new blood vessel growth from endothelial cells. In fibroplasia and granulation tissue formation, fibroblasts exert collagen and fibronectin to form a new, provisional extracellular matrix. Subsequently epithelial cells crawl across the wound bed to cover it and the wound is contracted by myofibroblasts, which grip the wound edges and undergo contraction using a mechanism similar to that in smooth muscle cells.

*Catharanthus roseus *L (apocyanaceae) also known as *Vinca Rosea*, is native to the Caribbean Basin and has historically been used to treat a wide assortment of diseases. European herbalists used the plant for conditions as varied as headache to a folk remedy for diabetes. It has more than 400 known alkaloids, some of which are approved as antineoplastic agents to treat leukemia, Hodgkin's disease, malignant lymphomas, neuroblastoma, rhabdomyosarcoma, Wilms' tumor, and other cancers. Its vasodilating and memory-enhancing properties have been shown to alleviate vascular dementia and Alzheimer's disease [3,4]. The two classes of active compounds in *Vinca *are alkaloids and tannins. The major alkaloid is vincamine and its closely related semi-synthetic derivative widely used as a medicinal agent, known as ethyl-apovincaminate or vinpocetine, has vasodilating, blood thinning, hypoglycemic and memory-enhancing actions [5,6]. The extracts of *Vinca *have demonstrated significant anticancer activity against numerous cell types [7].

Extracts from the dried or wet flowers and leaves of plants are applied as a paste on wounds in some rural communities. The fresh juice from the flowers of *C. roseus *made into a tea has been used by Ayurvedic physicians in India for external use to treat skin problems, dermatitis, eczema and acne. There is no previous report on wound healing activities of *C. roseus *in literature to the best of our knowledge and in this paper, we report for the first time, the efficacy of *C. roseus *flower extract in the treatment of wounds.

## Methods

### Plant material and extract preparation

The flowers of *C. roseus *were collected locally in April 2006 and identified by the plant taxonomist and curator, National Herbarium of Trinidad and Tobago, The University of the West Indies, St. Augustine, Trinidad and a voucher specimen was also deposited at the herbarium (specimen number: 36458). The fresh flowers were shade dried and ground into a powder using an electric blender. The fine powder (50 g) was suspended in 100 ml of ethanol for 20 hours at room temperature. The mixture was filtered using a fine muslin cloth followed by filter paper (Whatman No: 1). The filtrate was placed in a water bath to dry at 40°C and the clear residue was used for the study. The extract was subjected to preliminary phytochemical tests.

### Animals

The study was approved by the Ethics Committee for animal experimentation (AHC06/07/1), The Faculty of Medical Sciences, The University of the West Indies, St. Augustine

Healthy inbred gender-matched Sprague Dawley rats weighing 200–220 g were used for the study. They were individually housed and maintained on normal food and water *ad libitum*. Animals were periodically weighed before and after the experiment. The rats were anaesthetized prior to and during infliction of the experimental wounds. The surgical interventions were carried out under sterile conditions using ketamine anaesthesia (120 mg/kg). Animals were closely observed for any infection and those which showed signs of infection were separated and excluded from the study and replaced.

An acute toxicity study was conducted for the extract by the stair-case method [8]. The animals were fed with increasing doses of (1, 2, 4, and 8 g/kg body weight).

### Wound-healing activity

Excision, incision and dead space wound models were used to evaluate the wound-healing activity of *C. roseus*

### Excision wound model

Animals were anaesthetized prior to and during creation of the wounds. The rats were inflicted with excision wounds as described by Morton and Malon [9]. The dorsal fur of the animals was shaved with an electric clipper and the anticipated area of the wound to be created was outlined on the back of the animals with methylene blue using a circular stainless steel stencil. A full thickness of the excision wound of 2.5 cm (circular area = 300mm^2^) in length and 0.2 cm depth was created along the markings using toothed forceps, a surgical blade and pointed scissors. The entire wound was left open [10]. The animals were divided into two groups of 6 each. Group 1 animals were topically treated with the carboxymethyl cellulose (100 mg/kg/day) as a placebo control. The animals of group 2 were topically treated with the ethanol extract of *C. roseus *at a dose of 100 mg/kg/day) till complete epithelization. The wound closure rate was assessed by tracing the wound on days 1, 5 and 15 post-wounding using transparency paper and a permanent marker. The wound areas recorded were measured using a graph paper. Number of days required for falling of eschar without any residual raw wound gave the period of epithelization.

### Incision wound model

As with the above model rats were anaesthetized prior to and during creation of the wound. The dorsal fur of the animals was shaved with an electric clipper. A longitudinal paravertebral incision, six centimeters in length was made through the skin and cutaneous muscle on the back as described by Ehrlich and Hunt et al. [11]. After the incision, surgical sutures were applied to the parted skin at intervals of one centimetre. The wounds were left undressed. The rats were given flower extract (dissolved in drinking water) orally at a dose of 100 mg kg^-1 ^day^-1^. The controls were given with normal saline. The sutures were removed on the 8^th ^post wound day and the treatment was continued. The skin-breaking strength was measured on the 10^th ^day by the method described by Lee [12].

### Dead space wound model

Dead space wounds were inflicted by implanting two sterilized cotton pellets (10 mg), one on either side of in the lumbar region on the ventral surface of each rat. On the 10^th ^postwounding day, the granulation tissue formed on the implanted cotton pellet was carefully removed. The wet weight of the granulation tissue was noted. These granulation tissues were dried at 60°C for 12 hours, and weighed, and the weight was recorded. To the dried tissue added 5 ml 6 N HCl and kept at 110°C for 24 hours. The neutralized acid hydrolysate of the dry tissue was used for the determination of hydroxyproline [13].

### Determination of wound breaking strength

The anesthetized animal was secured to the table, and a line was drawn on either side of the wound 3 mm away from the line. This line was gripped using forceps one at each end opposed to each other. One of the forceps was supported firmly, whereas the other was connected to a freely suspended light weight metal plate. Weight was added slowly and the gradual increase in weight, pulling apart the wound edges. As the wound just opened up, addition of weight was stopped and the weights added was noted as a measure of breaking strength in grams. Three readings were recorded for a given incision wound, and the procedure was repeated on the contralateral wound. The mean reading for the group was taken as an individual value of breaking strength. The mean value gives the breaking strength for a given group

### Estimation of Hydroxyproline

Hydroxyproline present in the acid hydrolysate of granulation tissue oxidized by sodium peroxide in the presence of copper sulfate, when complexed with para-dimethylaminobezaldehyde, develops a pink color that was measured at 540 nm using colorimetry

### Phytochemical screening methods

Test for saponins: Boiled 300 mg of extract with 5 ml water for two minutes. Mixture was cooled and mixed vigorously and left it for three minutes. The formation frothing indicates the presence of saponins.

Test for tannins: To an aliquot of the extract added sodium chloride to make to 2% strength. Filtered and mixed with 1% gelatin solution. Precipitation indicates the presence of tannins.

Test for Triterpenes: 300 mg of extract mixed with 5 ml chloroform and warmed for 30 minutes. The chloroform solution is then treated with a small volume of concentrated sulphuric acid and mixed properly. The appearance of red color indicates the presence of triterpenes.

Test for alkaloids: 300 mg of extract was digested with 2 M HCl. Acidic filtrate was mixed with amyl alcohol at room temperature, and examined the alcoholic layer for the pink colour which indicates the presence of alkaloids.

Test for flavonoids: The presence of flavonoids was determined using 1% aluminum chloride solution in methanol, concentrated HCl, magnesium turnins, and potassium hydroxide solution.

The thin layer chromatography of the ethanol extract was done using following medium as mobile phase:

Petroleum ether: ethyl acetate (4:1 × 1 vol/vol)

Chloroform: methanol (4:1 × 1 vol/vol)

Chloroform: ethanol (1:1 × 1 vol/vol)

### Antimicrobial activity

*Pseudomonas aeruginosa, Beta-hemolytic streptococci, Enterobacter agglumerans and Staphylococcus aureus *were the organisms tested. The bacterial strains were obtained from fresh colonies grown on Mac Conkey and blood agar plates. The sensitivity testing was done using Muller Hinton Agar plates. Known volume of bacterial suspension was transferred to each microplate well. Ten microlitres of *C. roseus *extract dissolved in deionised water (200 μg/ml) was added to the microplate wells and incubated at 35–37°c for 18–20 h. Results were analyzed visually on the basis of turbid zone of inhibition. [+ = bacterium colonies deposited in the bottom of the well, ++ = turbidity with bacterium colonies being deposited, +++ = light turbidity, and ++++ = total growth inhibition].

### Statistical analysis

Results, expressed as mean ± SD were evaluated using Student's t-test and significance was set at p < 0.05.

## Results

In acute toxicity studies, the rats of either sex were fed with increasing doses (1, 2, 4 and 8 g/Kg body weight) of ethanol extract for 14 days. The doses up to 4 g/kg body weight did not produce any signs of toxicity and mortality. The animals were physically active and were consuming food and water in a regular way. We did not notice any abnormal behavior even with a dose of 8 g/kg body weight.

The significant increase in the wound-healing activity was observed in the animals treated with the *C. roseus *extract compared with those who received the placebo control treatments. Table 1 shows the effects of the ethanolic extract *C. roseus *flower administered orally at a dose of 100 mg kg^-1 ^day^-1 ^for 10 days on wound healing activity in rats inflicted with incision wound. In the incision wound model, a significant increase in the wound breaking strength (445.0 ± 4.43 g) was observed when compared with the controls. In the excision wound model, *C. roseus *treated animals showed a significant reduction in the wound area (p < 0.001) and epithelization period (Table 2). In the dead space wound model (Table 3), the ethanol extract-treated animals showed significantly increased levels of hydroxyproline content (p < 0.05) as compared with the control group of animals. A significant increase was observed in the weight (p < 0.001) of the granulation tissue in the animals treated with the extract.

**Table 1:** Wound healing effect of *C. roseus *in Incision wound model

Parameter	Placebo control	Experimental
Skin breaking strength (g)	319.13 ± 3.23	445.0 ± 4.43**

*N *= 6, Values are expressed as mean ± SD

*p < 0.05 and **p < 0.001 vs. control. Independent *t*-test

**Table 2:** Wound healing effect of *C. roseus in *Excision wound model

Parameter	Placebo control	Experimental
Wound area (mm^2^):		
Day 1	220.3 ± 23.80	222.50 ± 14.7
Day 5	173.6 ± 22.8	163.16 ± 31.58
Day 15	131.8 ± 25.90	65.40 ± 21.8 **
Period of epithelization (day)	14.2 ± 0.10	10.20 ± 0.13**

*N *= 6, Values are expressed as mean ± SD

**P < 0.001 vs. control. Independent *t*-test

**Table 3:** Wound healing effect of *C. roseus *in Dead space wound model

Parameter	Placebo control	Experimental
Wet weight of the granulation tissue (mg/100 g rat)	79.3 ± 19.20	98.60 ± 18.20*
Dry weight of the granulation tissue (mg/100 g rat)	7.4 ± 1.22	12.50 ± 1.90*
Hydroxyproline (mg/g tissue)	24.2 ± 6.11	63.00 ± 28.85*

*N *= 6, Values are expressed as mean ± SD

*P < 0.05 and vs. control. Independent *t*-test

The qualitative tests used to identify phytochemical constituents of the *C. roseus *showed the presence of triterpenoids, tannins and alkaloids. The thin layer chromatography of the ethanol extract showed 6 spots using petroleum ether: ethyl acetate (4:1 × 1 vol/vol) as mobile phase. The thin layer chromatography of the extract exhibited 7 spots using chloroform: methanol (4:1 × 1 vol/vol) as mobile phase. The extract showed 6 spots using chloroform: ethanol (2:1 × 1 vol/vol) as mobile phase. The specific staining of the thin layer slides showed the presence of alkaloids, triterpenoids and tannins. The extract tested at a concentration 200 μg/ml showed +++ pattern against the microbial organisms *P. aeruginosa*, and *S. aureus*. However, the Beta hemolytic streptococci and *Enterobacter agglumerans *were resistant against the extract that was indicated by abundant bacterium colonies deposited in the well [+] (Table 4).

**Table 4:** Antimicrobial activity of the *C. roseus *extract

Bacteria	Turbidity patterns
*Pseudomonas aeruginosa*,	*+++*
*Beta-hemolytic streptococci*	*+*
*Enterobacter agglumerans*	*+*
*Stphylococcus aureus*	*+++*

## Discussion

Wound healing is a complex and dynamic process of restoring cellular structures and tissue layers in damaged tissue as closely as possible to its normal state. Wound contracture is a process that occurs throughout the healing process, commencing in the fibroblastic stage whereby the area of the wound undergoes shrinkage. It has 3 phases; inflammatory, proliferative and maturational and is dependent upon the type and extent of damage, the general state of the host's health and the ability of the tissue to repair. The inflammatory phase is characterized by hemostasis and inflammation, followed by epithelization, angiogenesis, and collagen deposition in the proliferative phase. In the maturational phase, the final phase of wound healing the wound undergoes contraction resulting in a smaller amount of apparent scar tissue.

Granulation tissue formed in the final part of the proliferative phase is primarily composed of fibroblasts, collagen, edema, and new small blood vessels. The increase in dry granulation tissue weight in the test treated animals suggests higher protein content. The ethanol extract of *C. roseus *demonstrated a significant increase in the hydroxyproline content of the granulation tissue indicating increased collagen turnover. Collagen, the major component which strengthens and supports extra cellular tissue is composed of the amino acid, hydroxyproline, which has been used as a biochemical marker for tissue collagen [14].

The preliminary phytochemical analysis of the flower extract showed the presence of tannins, triterpenoids and alkaloids. Any one of the observed phytochemical constituents present in *C. roseus *may be responsible for the wound healing activity. Recent studies have shown that phytochemical constituents like flavanoids [15] and triterpenoids [16] are known to promote the wound-healing process mainly due to their astringent and antimicrobial properties, which appear to be responsible for wound contraction and increased rate of epithelialisation. Our earlier studies showed the presence of triterpenoids which were responsible for the effective wound healing activity of *Cecropia peltata *[17] and *Pentas lanceolata *[18].

The wound-healing property of *C. roseus *may be attributed to the phytoconstituents present in the plant, and the quicker process of wound healing could be a function of either the individual or the additive effects of the phytoconstituents. The early tissue approximation and increased tensile strength of the incision wound observed in our study may have been contributed by the tannin phytoconstituent of *C. roseus *from the astringent effect which has been reported elsewhere [19] Further phytochemical studies are in progress to isolate, characterize and identify the specific active compounds in this plant responsible for wound healing activity. Electron microscopic examination will yield the effect of the extract on angiogenesis, epithelialisation or collagen deposition. We plan to conduct additional studies to examine the constituent phytochemical constituents which contribute to the pharmacological activity of *C. roseus*. Wound healing activity of plant extracts may also be subsequent to an associated antimicrobial effect [20]. Our further studies will explore the antimicrobial effect of the extract and the specific phase of wound healing using electron microscopy.

## Conclusion

The present study has demonstrated that an ethanol extract of *C. roseus *flower has properties that render it capable of promoting accelerated wound healing activity compared with placebo controls. Wound contraction, increased tensile strength, increased hydroxyproline content and antimicrobial activity support further evaluation of *C. roseus *in the topical treatment and management of wounds.

## Competing interests

The author(s) declare that they have no competing interests.

## Authors' contributions

SN designed and conducted the work

LMP was responsible for pharmacological aspects of the experiments

All authors read and approved the final manuscript

## Pre-publication history

The pre-publication history for this paper can be accessed here:


